# Roux-en-Y gastric bypass and sleeve gastrectomy as revisional bariatric procedures after adjustable gastric banding: a retrospective cohort study

**DOI:** 10.1007/s00423-023-03174-y

**Published:** 2023-11-21

**Authors:** Hugo Santos-Sousa, Jorge Nogueiro, Luis Lindeza, Maria Neves Carmona, Filipe Amorim-Cruz, Fernando Resende, André Costa-Pinho, John Preto, Bernardo Sousa-Pinto, Silvestre Carneiro, Eduardo Lima-da-Costa

**Affiliations:** 1 Faculty of Medicine, University of Porto - Alameda Prof.Hernâni Monteiro, 4200-319 Porto, Portugal; 2 Obesity Integrated Responsibility Unit (CRI-O), São João University Medical Center, Alameda Prof. Hernâni Monteiro, 4200- 319 Porto, Portugal; 3 Surgery Department, São João University Medical Center, Alameda Prof. Hernâni Monteiro, 4200-319 Porto, Portugal; 4 MEDCIDS - Department of Community Medicine, Information and Health Decision Sciences, Faculty of Medicine, Rua Dr. Plácido da Costa, 4200-450 Porto, Portugal; 5 CINTESIS - Center for Health Technologies and Services Research, University of Porto, Rua Dr. Plácido da Costa, 4200-450 Porto, Portugal

**Keywords:** Revisional bariatric surgery, Adjustable gastric band, Roux-en-Y gastric bypass, Sleeve gastrectomy, Obesity

## Abstract

**Introduction:**

The frequency of revisional bariatric surgery is increasing, but its effectiveness and safety are not yet fully established. The aim of our study was to compare short-term outcomes of primary (pRYGB and pSG) and revisional bariatric surgeries (rRYGB and rSG).

**Methods:**

We performed a retrospective cohort study assessing all patients submitted to primary and revisional (after a failed AGB) RYGB and SG in 2019. Each patient was followed-up at 6 months and 12 months after surgery. We compared pRYGB vs. rRYGB, pSG vs. rSG and rRYGB vs. rSG on weight loss, surgical complications, and resolution of comorbidities.

**Results:**

We assessed 494 patients, of which 18.8% had undergone a revisional procedure. Higher weight loss at 6 and 12 months was observed in patients undergoing primary vs. revisional procedures. Patients submitted to rRYGB lost more weight than those with rSG (%EWL 12 months = 82.6% vs. 69.0%, *p* < 0.001). Regarding the resolution of obesity-related comorbidities, diabetes resolution was more frequent in pRYGB than rRYGB (54.2% vs. 25.0%; *p* = 0.038). Also, 41.7% of the patients who underwent rRYGB had dyslipidemia resolution vs. 0% from the rSG group (*p* = 0.035). Dyslipidemia resolution was also more common in pSG vs. rSG (68.6% vs. 0.0%; *p* = 0.001). No significant differences in surgical complications were found.

**Conclusion:**

Revisional bariatric surgery is effective and safe treating obesity and related comorbidities after AGB. Primary procedures appear to be associated with better weight loss outcomes. Further prospective studies are needed to better understand the role of revisional bariatric surgery.

## Introduction

Obesity is a complex multifactorial disease, whose prevalence is increasing steadily in the last decades [1]. The World Health Organization (WHO) identifies obesity as abnormal or excessive fat accumulation with a body mass index (BMI) of 30 kg/m^2^ or higher, representing a health challenge. It affects almost all physiological functions of the body, and it increases the risk for developing multiple comorbidities, such as diabetes mellitus type 2 (T2DM), cardiovascular disease, cancer, osteoarthritis, obstructive sleep apnea (OSA), and poor mental health, thereby contributing to a decline in quality of life, work productivity as well as to increased healthcare costs [2].

Bariatric surgery is regarded as the most effective intervention for achieving substantial and long-lasting weight loss and resolution of obesity associated comorbidities, exceeding the results obtained with medical treatment [3]. The laparoscopic implantation of an adjustable gastric banding (AGB) was first described in 1993 and it became one of the most common bariatric procedures in the world [4]. Its popularity was due to the remarkable safety profile and low initial morbidity rate [5]. The AGB has a balloon which can be inflated, constricting the stomach in order to reduce the patient’s oral intake [6]. Complications of AGB were initially believed to be minor and rare. However, long-term studies have progressively shown late-onset complications that lead to revisional surgeries [5]. The high complication rate (including hardware malfunctions with the band, tubing, or access port; esophageal motility disorders; adverse gastrointestinal symptoms; and psychological intolerance to the band) negatively impacts patients’ satisfaction and quality of life [7, 8]. Moreover, a significant fraction of patients submitted to AGB fail to lose weight or have weight regain, frequently requiring conversion to other bariatric procedures [5]. Recent studies have reported long-term reoperations in 31–80% of patients following failed AGB [9].

Nowadays, Roux-en-Y gastric bypass (RYGB) and sleeve gastrectomy (SG) are the two most commonly performed bariatric procedures [10]. RYGB has shown better outcomes regarding the resolution of obesity-related comorbidities [11, 12]. However, SG is still the most frequent bariatric surgery performed because it is technically easier and faster to perform, resulting in fewer postoperative complication and reoperation rates and in similar excess weight reduction [13, 14].

Revisional bariatric surgeries are becoming increasingly common due to inadequate weight reduction, weight regain, and postoperative morbidity [8, 15, 16]. The most successful conversion strategy relies on selecting the most appropriate revisional intervention, depending mostly on the indications for revision themselves [8]. Despite the growing demand for revisional procedures, there is still some controversy on their safety and efficacy [16]. Also, there is insufficient evidence on which procedure is more suitable for each patient. Despite the lack of standardized guidelines for the conversion strategy from AGB, RYGB and SG are the most frequent procedures applied [17].

The aim of our study is to compare short-term outcomes of primary (pRYGB and pSG) vs. revisional bariatric surgeries (rRYGB and rSG), and between different types of revisional interventions on weight loss outcomes, surgical complications, and resolution of obesity-related comorbidities.

## Materials and methods

### Participants and setting

This is a retrospective cohort study assessing a consecutive sample of all adult (age > 18 years) patients submitted to either primary or revisional RYGB or SG between January to December 2019 in a single tertiary hospital in Northern Portugal. We excluded patients submitted to a revisional surgery whose primary procedure was not the implantation of a laparoscopic AGB.

In our institution, all patients are evaluated multidisciplinary (surgery, endocrinology, nutrition, psychology/psychiatry, and anesthesiology) and per-protocol, all patients are submitted to extensive blood and urine analysis, upper endoscopy and abdominal ultrasonography, and eligibility criteria are defined accordingly to ASMBS and IFSO criteria. Gastric bypass is suggested for individuals dealing with hiatal hernias, GERD, or esophagitis, as well as patients with other comorbidities that will benefit from this procedure, like diabetes or psoriasis. On the other hand, sleeve gastrectomy is proposed to patients with specific diseases, like inflammatory bowel disease, and those with unamenable risk factors for gastric cancer, like persistent Helicobacter Pylori infection or familiar history of gastric cancer. Patient opinion and preferences are other important factor to take in account. Challenging cases are discussed in a multidisciplinary meeting held once a week.

### Variables

From each patient, we retrieved information on demographic characteristics, obesity-related comorbidities, previous surgeries, and surgical complications. Anthropometric data, metabolic parameters and clinical outcomes, medication, and nutritional supplements from the included patients were retrospectively collected from their electronic medical charts. We reviewed the data from the preoperative period, at the date of the surgery, and postoperatively at 6 and 12 months.

The revision from AGB was a 2-step operation, consisting of band removal followed (at a subsequent date) by a conversion operation, either RYGB or SG. RYGB was performed as a standardized technique with a small gastric pouch, a biliopancreatic limb of 100 cm, and an alimentary limb of 120 cm. SG was performed using a 54-Fr Fouchet tube, sectioning the stomach from the gastric antrum to the angle of His. The choice of bariatric procedure was made individually regarding patient characteristics, comorbidities, previous treatments, preference, and surgeon recommendation.

Weight loss was quantified as the percentage of total weight loss (%TWL) and percentage of excess weight loss (%EWL). Successful weight loss after surgery was defined if patients completed the following criteria at 12 months: %TWL ≥ 20%, %EWL ≥ 50%, and BMI < 35 kg/m^2^. The criteria used to evaluate the resolution of T2DM, dyslipidemia, arterial hypertension (HTN), and OSA were adapted from the American Society for Metabolic and Bariatric Surgery (ASMBS) consensus statement “Standardized outcomes reporting in metabolic and bariatric surgery” [18]. Improvement of psychiatric disorders, gastroesophageal reflux disease, and osteoarticular pathologies were not evaluated in our series. Postoperative early-onset complications until 90 days after the date of the surgery were also evaluated.

### Statistical analysis

Categorical variables are presented with absolute frequencies and percentages and continuous variables are presented with means ± standard deviations (SD) or with medians and interquartile ranges (IQR), depending on data distribution. The Chi-Square test was used to compare categorical variables. The independent samples t-test was used for the continuous variables with a normal distribution and the Mann–Whitney U test was applied for the continuous variables which do not follow a normal distribution. A *p*-value < 0.05 was considered statistically significant. IBM SPSS Statistics 26® (SPPS Inc., Chicago, Ill., USA) was used to perform the statistical analysis.

## Results

**Fig. 1:**
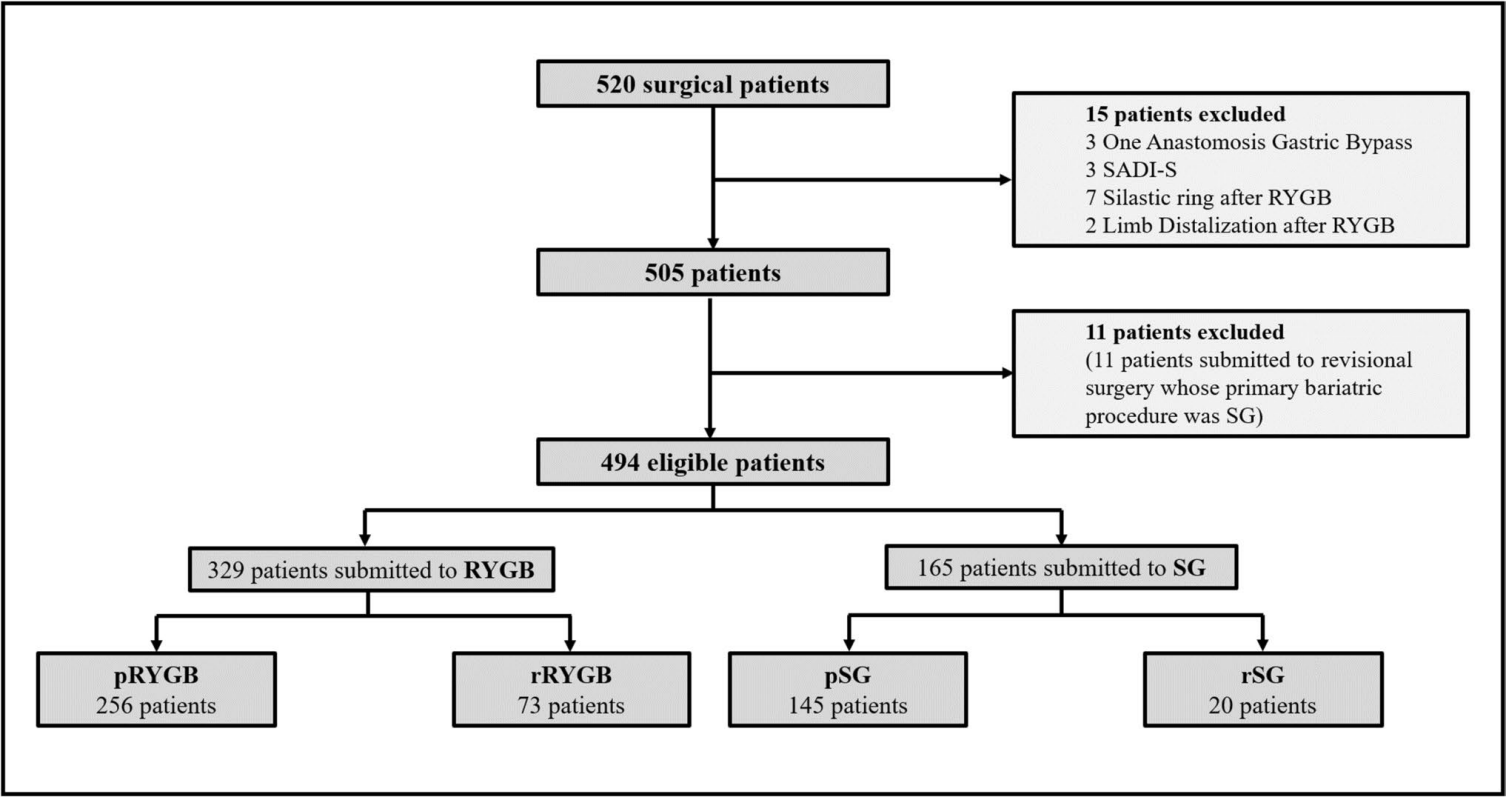
Flowchart of patients’ eligibility

Of the 520 patients submitted to bariatric surgery in 2019 at our hospital, 494 patients met the eligibility criteria and were included in this study (Fig. 1). Ninety-three (18.8%) of the eligible participants underwent a revisional bariatric surgery after a failed AGB. RYGB was the most common bariatric procedure: 329 (66.6%) patients were submitted to RYGB, 73 (22.2%) of them were patients undergoing rRYGB. Of the 165 patients undergoing SG, 20 (12.1%) had this procedure as a revisional bariatric surgery.

The characteristics of the participants are presented in Table 1. Median age on presentation was 45.0 years for pRYGB and 49.0 for rRYGB (*p* = 0.001). Median age was 44.0 years for pSG vs. 51.0 years for rSG (*p* = 0.002). One pSG was performed as an open surgery, associated to other procedure (bowel reconstruction). As for the remaining bariatric procedures, they were done laparoscopically, not requiring conversion to open surgery. There was no mortality in either group. There were no differences regarding pre-operative BMI and pre-operative comorbidities (30 Clavien-Dindo II; 1 Clavien-Dindo IIIb). Every patient completed the 12-month follow-up of our study.

**Table 1:** Demographic characteristics

	pRYGB (*n* = 256)	pSG (*n* = 145)	rRYGB (*n* = 73)	rSG (*n* = 20)	*p-value*		
					pRYGB vs*.* rRYGB	pSG vs*.* rSG	rRYGB vs*.* rSG
Age (years); median [IQR]	45.00 [IQR 38.25–53.00]	44.00 [IQR 35.50–54.00]	49.00 [IQR 42.00–58.00]	51.00 [IQR 44.00–58.25]	0.001	0.015	0.653
Female; *n* (%)	224 (87.5%)	103 (71.0%)	67 (91.8%)	17 (85.0%)	0.313	3.189	0.399
Preoperative BMI (kg/m^2^); median [IQR]	42.27 [IQR 39.30–45.20]	43.78 [IQR 39.93–49.12]	42.67 [IQR 39.32–45.46]	44.63 [IQR 39.61–49.04]	0.760	0.759	0.180
Preoperative comorbidities							
HTN; *n* (%)	119 (46.5%)	72 (49.7%)	36 (49.3%)	10 (50.0%)	0.669	0.977	0.957
T2DM; *n* (%)	74 (28.9%)	33 (22.8%)	21 (28.8%)	3 (15.0%)	0.982	0.570	0.213
Dyslipidemia; *n* (%)	119 (46.5%)	69 (47.6%)	33 (45.2%)	10 (50.0%)	0.847	0.839	0.102
OSA; *n* (%)	48 (14.6%)	29 (20.0%)	10 (3.0%)	6 (30.0%)	0.318	0.380	0.703

*pRYGB*, primary Roux-en-Y gastric bypass; *pSG*, primary sleeve gastrectomy; *rRYGB*, revisional Roux-en-Y gastric bypass; *rSG*, revisional sleeve gastrectomy; *BMI*, body mass index; *HTN*, arterial hypertension; *T2DM*, diabetes mellitus type 2; *OSA*, obstructive sleep apnea

**Fig. 2:**
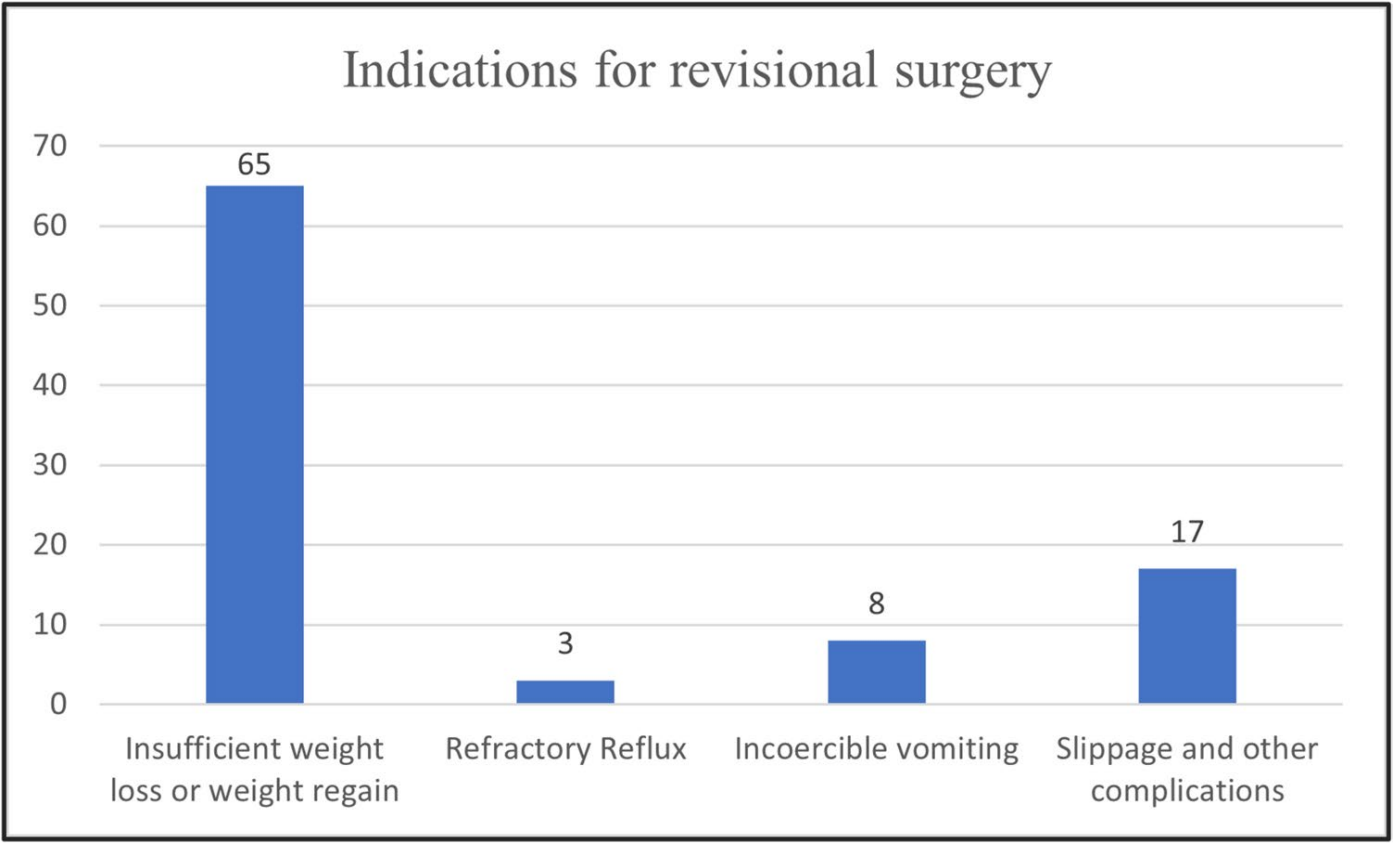
Indications for revisional surgery

Figure 2 displays the indications for revisional bariatric surgery. Insufficient weight loss was the main reason for revisional surgery: 65 (69.9%) patients removed the AGB due to inadequate weight loss or weight regain. Slippage and other complications (17 patients; 18.8%), incoercible vomiting (8 patients; 8.6%), and refractory reflux (3 patients; 3.2%) were other indications reported in our series.

### pRYGB vs. rRYGB

Weight loss outcomes are presented in Table 2. We found significant higher %TWL and %EWL and a lower BMI at 6 and 12 months in the pRYGB group. The frequency of patients reaching the qualitative criteria of %TWL ≥ 20%, %EWL ≥ 50%, and BMI < 35 kg/m^2^ did not differ between the two groups.

**Table 2:** Weight loss outcomes

*p-value*							
	pRYGB	pSG	rRYGB	rSG	pRYGB vs*.* rRYGB	pSG vs*.* rSG	rRYGB vs*.* rSG
*Quantitative parameters*							
%EWL at 6 months; median [IQR]	71.69 [IQR 71.40–87.22]	62.56 [IQR 50.69–76.36]	61.12 [IQR 51.99–77.25]	51.21 [IQR 41.93–63.39]	**0.004**	**0.007**	**0.022**
%TWL at 6 months; mean ± SD	28.71 ± 6.09	27.31 ± 6.59	24.80 ± 6.61	21.35 ± 4.74	**< 0.001**	** < 0.001**	**0.025**
BMI at 6 months (kg/m^2^); median [IQR]	30.11 [IQR 27.73–32.79]	32.04 [IQR 28.66–35.13]	31.68 [IQR 28.42–34.28]	34.68 [IQR 29.29–38.03]	**0.042**	0.080	**0.035**
%EWL at 12 months; median [IQR]	87.50 [IQR 78.08–101.06]	77.16 [IQR 63.80–94.08]	82.64 [IQR 68.01–93.62]	69.01 [IQR 51.20–74.82]	**0.040**	**0.007**	**< 0.001**
%TWL at 12 months; mean ± SD	36.09 ± 7.24	33.55 ± 8.38	32.41 ± 7.33	26.77 ± 6.45	**0.001**	**0.003**	**0.005**
BMI at 12 months (kg/m^2^); median [IQR]	27.16 [IQR 24.84–29.20]	29.45 [IQR 25.98–33.17]	28.37 [IQR 25.64–31.15]	34.16 [IQR 29.38–36.20]	**0.047**	**0.012**	**< 0.001**
*Qualitative parameters*							
BMI < 35 kg/m^2^ at 12 months; *n* (%)	209 (97.2%)	88 (84.6%)	59 (98.3%)	9 (60.0%)	1.000	**0.033**	** < 0.001**
%EWL > 50% at 12 months; *n* (%)	211 (98.1%)	96 (92.3%)	56 (93.3%)	12 (80.0%)	0.072	0.143	0.138
%TWL > 20% at 12 months; *n* (%)	211 (98.1%)	99 (95.2%)	57 (95.0%)	12 (80.0%)	0.178	0.062	0.090

*pRYGB*, primary Roux-en-Y gastric bypass; *pSG*, primary sleeve gastrectomy; *rRYGB*, revisional Roux-en-Y gastric bypass; *rSG*, revisional sleeve gastrectomy; *%EWL*, percentage of excess weight loss; *%TWL*, percentage of total weight loss; *BMI*, body mass index; bold entries include values of statistically significance

Table 3 presents the results concerning morbidity and mortality. Operative time was shorter in the pRYGB group (median: 90.00 min [IQR 76.00–109.00] vs. 117.00 min [IQR 96.75–145.50],* p* < 0.001). Fourteen (5.5%) patients in the pRYGB group developed postoperative complications vs. 4 (5.5%) patients in the rRYGB group (*p* = 1.000).

**Table 3:** Morbidity and mortality

*p-value*							
	pRYGB	pSG	rRYGB	rSG	pRYGB vs*.* rRYGB	pSG vs*.* rSG	rRYGB vs*.* rSG
*Overall complications*							
Early complications; *n* (%)	14 (5.5%)	11 (7.6%)	4 (5.5%)	2 (10.0%)	1.000	0.660	0.606
Mortality; *n* (%)	0 (0.0%)	0 (0.0%)	0 (0.0%)	0 (0.0%)	-	-	-
Operative time (minutes); median [IQR]	90.00 [IQR 76.00 -109.00]	62.50 [IQR 50.75 -83.50]	117.00 [IQR 96.75–145.50]	86.00 [IQR 59.00–129.00]	** < 0.001**	**0.016**	**0.020**

*pRYGB*, primary Roux-en-Y gastric bypass; *pSG*, primary sleeve gastrectomy; *rRYGB*, revisional Roux-en-Y gastric bypass; *rSG*, revisional sleeve gastrectomy; bold entries include values of statistically significance

Concerning the resolution of obesity-related comorbidities, 32 of the 59 (54.2%) valid cases (with preoperative comorbidities) submitted to pRYGB and 4/ out of 16 (25.0%) patients who underwent rRYGB had a T2DM resolution (*p* = 0.038) (Table 4). No significant differences were observed regarding the resolution of HTN, dyslipidemia, and OSA.

**Table 4:** Resolution of comorbidities

*p-value*							
Obesity-related comorbidity	pRYGB	pSG	rRYGB	rSG	pRYGB vs*.* rRYGB	pSG vs*.* rSG	rRYGB vs*.* rSG
HTN; *n*/*N* (%)	37/93 (39.8%)	20/40 (50,0%)	6/28 (21.4%)	1/7 (14.3%)	0.075	0.112	1.000
T2DM; *n*/*N* (%)	32/59 (54.2%)	14/21 (66.7%)	4/16 (25.0%)	0/2 (0.0%)	**0.038**	0.142	1.000
Dyslipidemia; *n*/*N* (%)	38/78 (48.7%)	24/35 (68.6%)	10/24 (41.7%)	0/8 (0.0%)	0.545	**0.001**	**0.035**
OSA; *n*/*N* (%)	16/29 (55.2%)	8/16 (50.0%)	3/6 (50.0%)	0/4 (0.0%)	1.000	0.117	0.200

*pRYGB*, primary Roux-en-Y gastric bypass; *pSG*, primary sleeve gastrectomy; *rRYGB*, revisional Roux-en-Y gastric bypass; *rSG*, revisional sleeve gastrectomy; *BMI*, body mass index; *HTN*, arterial hypertension; *T2DM*, diabetes mellitus type 2; *OSA*, obstructive sleep apnea, *n*, number of cases who had obesity-related comorbidities’ resolution; *N*, number of valid cases; bold entries include values of statistically significance

### pSG vs. rSG

The %TWL and %EWL at 6 and 12 months were higher in the pSG group than the rSG group. The BMI at 6 months was not statistically different between the two groups, but we found a statistically lower BMI at 12 months in the pSG group when compared to the rSG group (median: 29.45 [IQR 25.98–33.17] vs. 34.16 [IQR 29.38–36.20], *p* = 0.012). Also, at 12 months, 84.6% of the patients submitted to pSG had BMI < 35 kg/m^2^ vs*.* 60.0% of the rSG patients (*p* = 0.033). No significant differences were observed on the remaining qualitative criteria.

Operative time was longer in the rSG group (62.5 min [IQR 50.75–83.5] vs. 86.0 [IQR 59.00–129.00]; *p* = 0.016). Postoperative complications developed in 11 (7.6%) patients in the pSG group and 2 (10.0%) patients in the rSG group (*p* = 0.660).

Concerning the outcomes of comorbidities, 24 of the 35 (68.6%) valid cases in the pSG group and 0/8 (0.0%) patients in the rSG group had dyslipidemia resolution. No other significant differences were observed.

### rRYGB vs. rSG

We found statistically significant higher %TWL and %EWL and a lower BMI at 6 and 12 months in the rRYGB group when compared to the rSG group. Also, 98.3% of the patients submitted to rRYGB had BMI < 35 kg/m^2^ vs*.* 60% of the rSG patients (*p* < 0.001). No significant differences were observed on the remaining qualitative criteria, even though results tended to favor rRYGB.

Operative time was longer in the rRYGB group (90.00 [IQR 76.00–109.00] vs. 117.00 [IQR 96.75–145.50] *p* = 0.020). Four (5.5%) patients in the rRYGB group and 2 (10.0%) patients in the rSG group developed postoperative complications (*p* = 0.606).

Concerning the resolution of obesity-related comorbidities, 10/24 (41.7%) patients submitted to rRYGB and 0/8 (0.0%) patients who underwent rSG had the resolution of dyslipidemia (*p* = 0.035). No significant differences were observed regarding the resolution of HTN, T2DM, and OSA.

## Discussion

AGB used to be the most common bariatric surgery performed worldwide due to its technical simplicity and low surgical risk [19]. However, it has been associated with high rates of complications requiring revision [5–7, 20]. According to our findings, weight regain, and insufficient weight loss were the most common indications for revisional bariatric surgery, accounting for more than two-thirds of patients submitted to revisional procedures. Other studies report similar findings—in a systematic review, Magouliotis et al. reported that among patients submitted to rRYGB and rSG, two-thirds had the indication of insufficient weight loss after AGB [20].

The present study demonstrates that both rRYGB and rSG are safe procedures. We did not find any significant difference between either the primary and revisional bariatric surgery groups or between the different revisional groups on the frequency of early complications (even though revisional bariatric interventions are technically more demanding and they are usually associated with higher intraoperative and perioperative risks than primary procedures) [9, 21, 22]. The absence of significant differences concerning postoperative complications can be explained by the surgeons’ expertise and the high number of bariatric procedures performed annually at our center. It is consensual in the literature that the experience of the surgical team, as well as the hospital volume, are associated with better outcomes for bariatric surgical procedures [23]. The ASMBS recommends that these revisional procedures should only be done by experienced bariatric surgeons in health care centers with the resources to manage these challenging patients and to provide early treatment to patients who potentially develop postoperative complications [24].

We have also found an increased operation time in the revisional bariatric procedures, which is reported in several studies [21, 22, 25]. rRYGB took longer than rSG, which is in accordance with several studies [20, 26]. This difference may be explained by the fact SG is a technically easier surgical approach [14].

Regarding the weight loss outcomes, we found significant differences at 6 and 12 months between the primary and revisional groups. Patients submitted to pRYGB lost more weight and displayed a better BMI than those who underwent rRYGB. The literature is almost unanimous stating that pRYGB has better weight loss outcomes than rRYGB [27, 28].

Similar conclusions can be drawn when comparing pSG with rSG. pSG group had better weight loss outcomes than rSG. There is insufficient evidence evaluating weight loss outcomes between pSG and rSG after AGB removal, but a few studies report similar weight loss outcomes after rSG when compared to pSG [29].

There is no consensus concerning the weight loss outcomes comparing both revisional procedures. Performing SG after a failed AGB is often criticized because they are both restrictive bariatric procedures, but many others state that the excision of the gastric fundus may have endocrine effects, reducing the ghrelin levels [8, 19]. Many studies report no difference between rRYGB and rSG [26], while other studies conclude that rRYGB has better weight loss outcomes than rSG and is the most appropriate revision procedure if the primary cause for AGB removal was inadequate weight loss [8]. At 6 and 12 months, rRYGB had consistently better weight loss outcomes than rSG. Also, we found that a higher percentage of patients submitted to rRYGB reached BMI < 35 kg/m^2^ compared to those undergoing rSG.

It has been established that revisional bariatric surgery plays a role in the improvement and remission of obesity-related comorbidities [24]. However, whether there are differences in the improvement of comorbidities between procedures is still controversial—most of the studies indicate that pRYGB is associated with higher improvement of comorbidities, [30, 31] but other studies found similar rates between pRYGB and rRYGB regarding obesity-related diseases’ resolution [27, 32]. In this study, we found that pRYGB was associated with significantly higher frequency of T2DM resolution, but such was not observed for other comorbidities. Similarly, we observed higher frequency of dyslipidemia resolution with pSG compared to rSG, but no significant differences were observed for other comorbidities. However, these results should be analyzed with caution, given that different indications are used for each procedure.

There is shortage of quality data in the literature comparing revisional procedures concerning resolution of preoperative comorbidities [33]. Although many studies draw recommendations of which revisional surgery is more appropriate based on the primary cause for AGB removal, there are few studies addressing which one is better for the treatment of obesity comorbidities. Our series showed better outcomes regarding the resolution of dyslipidemia in rRYGB patients than those who were submitted to rSG.

The results regarding resolution of obesity-related comorbidities must be interpreted with caution because there is a substantial number of cases missing, since our follow-up period included the COVID-19 pandemic lockdown, with many appointments being done by phone. It was difficult to accurately assess if the patients found the criteria to establish resolution of comorbidities.

Limitations of our work include its retrospective nature. This study was performed in a single institution. Nonetheless, this assures homogeneity in treatment plan, because all patients were evaluated and treated by the same multidisciplinary team. Furthermore, with a short follow-up time, it is difficult to extrapolate long-term outcomes. Also, due to the COVID-19 pandemic, some follow-up consultations were accomplished remotely by telephone. Hence, this study cannot be used to draw conclusions about the long-term expected weight loss or comorbidities’ resolution after revisional bariatric surgery without additional longitudinal study.

## Conclusion

Revisional bariatric surgery is a safe and effective option to treat obesity and related comorbidities. Our study showed poorer weight loss outcomes of revisional compared to primary RYGB and SG. RYGB was associated with better outcomes compared to SG concerning weight reduction when they are both used as conversion strategies following failed AGB. rSG is less effective than pSG and rRYGB on dyslipidemia’s resolution. The choice of the most suitable primary procedure for each patient is of paramount importance. Further clinical studies, with a prospective design and longer follow-up, are necessary to better understand the role of revisional bariatric surgery in the treatment of obesity.

## Data Availability

All the data supporting the findings of this study are available from the authors upon reasonable request.
